# Long Lasting Egocentric Disorientation Induced by Normal Sensori-Motor Spatial Interaction

**DOI:** 10.1371/journal.pone.0004465

**Published:** 2009-02-12

**Authors:** Eve Dupierrix, Michael Gresty, Théophile Ohlmann, Sylvie Chokron

**Affiliations:** 1 Laboratoire de Physiologie de la Perception et de l'Action (LPPA), CNRS, UMR 7152, Collège de France, Paris, France; 2 Laboratoire de Psychologie & Neuro-Cognition (LPNC), CNRS, UMR 5105, Grenoble, France; 3 ERT TREAT Vision, Fondation Ophtalmologique A. de Rothschild, Service de Neurologie, Paris, France; 4 MRC, Section of Neuro-Otology, Division of Neuroscience & Mental Health, Imperial College London, London, United Kingdom; Universidade Federal do Rio de Janeiro (UFRJ), Instituto de Biofísica da UFRJ, Brazil, Brazil

## Abstract

**Background:**

Perception of the cardinal directions of the body, right-left, up-down, ahead-behind, which appears so absolute and fundamental to the organisation of behaviour can in fact, be modified. Perhaps unsurprisingly, it has been shown that prolonged distorted perception of the orientation of body axes can be a consequence of disordered sensori-motor signals, including long-term prismatic adaptation and lesions of the central nervous system. We report the novel and surprising finding that a long-lasting distortion of perception of personal space can also be induced by an ecological pointing task without the artifice of distorting normal sensori-motor relationships.

**Methodology/Principal Findings:**

Twelve right-handed healthy adults performed the task of pointing with their arms, without vision, to indicate their subjective ‘straight ahead’, a task often used to assess the Egocentric Reference. This was performed before, immediately, and one day after a second task intended to ‘modulate’ perception of spatial direction. The ‘modulating’ task lasted 5 minutes and consisted of asking participants to point with the right finger to targets that appeared only in one (right or left) half of a computer screen. Estimates of the ‘straight-ahead’ during pre-test were accurate (inferior to 0.3 degrees deviation). Significantly, up to one day after performing the modulating task, the subjective ‘straight-ahead’ was deviated (by approximately 3.2 degrees) to the same side to which subjects had pointed to targets.

**Conclusion/Significance:**

These results reveal that the perception of directional axes for behaviour is readily influenced by interactions with the environment that involve no artificial distortion of normal sensori-motor-spatial relationships and does not necessarily conform to the cardinal directions as defined by the anatomy of orthostatic posture. We thus suggest that perceived space is a dynamic construction directly dependent upon our past experience about the direction and/or the localisation of our sensori-motor spatial interaction with environment.

## Introduction

Our perception of the cardinal directions of the body (right-left, up-down, ahead-behind), which appear to be so fundamental can in fact be modified. The primary direction of Straight-Ahead (SA) can be biased left or rightward in healthy humans by several sensori-motor manipulations [see 1 for review] including trunk rotation [2], neck muscle vibration [3], [4], optokinetic stimulation [5] or prismatic adaptation [6], [7], [8]. Perhaps less surprisingly, unilateral lesion producing Unilateral Spatial Neglect can also bias the perception of SA or left-right symmetry [9], [10], [11] so that when neglect patients are asked to point straight-ahead, they often show substantial deviations of the SA towards the side of the lesion. Thus, so far, the conditions that have been identified as distorting perceived body axes have involved disordered sensori-motor signals or central nervous system damage. With the exception of the demonstration by Hatada and collaborators [6] that long exposure to viewing through prisms can induce long-lasting changes in SA estimations, the various experimental sensori-motor manipulations explored in healthy adults have managed only to induce biases in the perception of body axes that last but a brief time.

In this study, we aimed to show that experience of a brief lateralized conventional sensori-motor task (Lateralized Pointing, LP) can result in a long-lasting ipsilateral bias in the perceived direction of the SA. The current LP task involved normally coordinated visuo-motor pointing, unlike prism adaptation in which normal sensori-motor directional correspondences were de-correlated (through a lateral shift of visual field). The experiment was suggested by the recent observation that LP task can elicit short-term biases in estimates of ‘centre’ in line bisection tasks [12].

Twelve right-handed blindfolded healthy volunteers undertook an arm pointing task in which they indicated the direction of their subjective SA with their left forefinger for half of the trials and their right one for the remaining half. This proprioceptive SA task was performed immediately before (‘pre-test’), immediately after (‘post-test’) and one day after (‘late test’) the 5-min LP task in which participants were asked to point their right arm towards visual targets that appeared briefly only in one hemispace (in the left field for half of the participants and in the right field for the remaining half). The subjective SA direction was assessed at each trial during the pre-, post-, and late-test sessions as the algebraic angular distance between the objective SA and the direction as indicated by the participant's forefinger.

## Results

The expected influence of the LP task on post- and late- test SA estimations compared to pre-test was examined by ANOVA with the LP Group as a between factor and the Session and the Hand used for the SA task as within factors. These analyses were separately performed on both the algebraic and absolute scores (measured to within half a degree). The algebraic values indicate the laterality of the SA estimation (i.e. to the left or right of the objective 0°) whereas absolute values reflect the accuracy for pointing SA, i.e. the amplitude of the subjective SA deviation compared to 0°, regardless of the laterality of the SA deviation.

**Figure 1:**
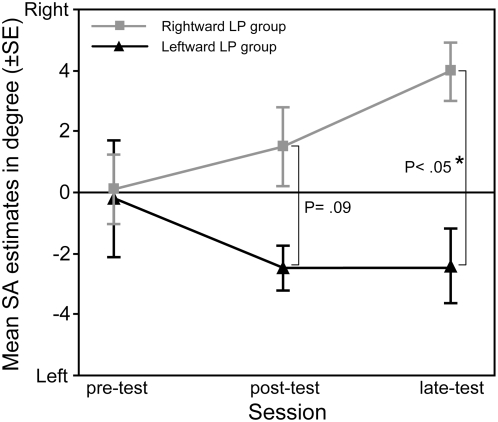
Mean deviation of subjective SA (±SE) as a function of Session and LP Group. Leftward and rightward deviations from the true SA were respectively coded as negative and positive values.

**Figure 2:**
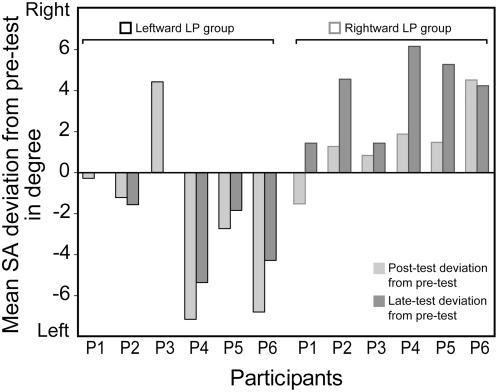
Short- and long-lasting influences of the LP task on SA for the six participants of each (leftward and rightward) LP group. The bars reflect the mean SA deviation at post-test and late-test compared to pre-test for each participant. Negative difference was interpreted as a leftward deviation as compared to pre-test, a positive difference as revealing a rightward deviation.

Table 1 provides the mean SA estimates as a function of the LP Group and the Session and Fig. 1 shows the subjective SA for each LP Group by Session. Compared to pre-test, a deviation of the SA position appears immediately and one day after the LP task, toward the right side for the rightward LP group (pre-test: *M* = +0.10°, SE = 1.15; post-test: *M* = +1.52°, SE = 1.30°; late-test: *M* = +3.96°, SE = 0.95°) and toward the left side for the leftward LP group (pre-test: *M* = −0.22°, SE = 1.91; post-test: *M* = −2.50°, SE = 0.72°; late-test: *M* = −2.42°, SE = 1.21°). The two way ANOVA (leftward *vs.* rightward LP Group x three sessions x two hands used for SA pointing) confirmed this observation by revealing a significant main effect of Group (F(1, 10) = 5.27, CMe = 43.52, p<.05) and an interaction of Group with Session (F(2, 20) = 8.19, CMe = 6.83, p<.005). No interaction of these effects with the hand used for the SA pointing was observed. The decomposition of the Group by Session interaction shows that the influence of the LP task was marginal at immediate post-test as compared to pre-test (F(1, 10) = 3.61, CMe = 11.33, p = .087) and significant for the late-test measurement (F(1, 20) = 25.21, CMe = 4.37, p<.001). As illustrated in the Fig. 2, the influence of the LP task was obvious in 4 of the 6 participants included in the leftward LP group and 5 of the 6 adults participated in the rightward LP task. Furthermore, as shown on the Fig. 1 (and observed in all participants except the sixth in the Fig. 2), there appears to be a difference for the rightward LP group between the post- and late-test. The Newman-Keuls Post-hoc procedure [13] revealed that this difference to be significant (p<.05).

**Table 1:** Mean SA estimates assessed for each LP group as a function of the Session.

LP group	Session	Algebraic score (SE)	Absolute score (SE)
Leftward	pre-test	−0.22 (1.91)	4.44 (0.26)
	post-test	−2.50 (0.72)	3.59 (0.30)
	late-test	−2.42 (1.21)	4.18 (0.31)
Rightward	pre-test	+0.10 (1.15)	3.13 (0.32)
	post-test	+1.52 (1.30)	3.48 (0.30)
	late-test	+3.96 (0.95)	5.04 (0.31)

Algebraic (and absolute) scores correspond to the algebraic (and absolute) deviations of the SA averaged across trials and participants.

The analysis conducted on absolute SA deviation did not reveal any significant effect.

## Discussion

The aim of the present study was to show that perceived personal space depends on interactions with environment that involve no artificial distortion of normal sensori-motor spatial relationships. Whichever hand was used for SA estimates, long-lasting lateral deviations were shown to the same side of our brief LP task, without change in accuracy. Theses findings demonstrate that experience of a lateralized, normal, visuo-motor coordination task can produce a subsequent long-lasting modulation in the perceived direction of body axes or in left-right spatial symmetry.

Given that the LP task probably affected both of the hands used to point SA, the post-test SA deviations were unlikely to result from muscular effort or modification in the sensation of limb position linked to LP task [14], [15], [16] since only the right hand was mobilized during the LP task. In the same vein, a muscular explanation cannot predict the long-lasting duration of the current effect and particularly, its increasing amplitude in the late-test (for the rightward group). Therefore, as shown by studies dealing with prismatic adaptation [6], [17], we believe that such a long lasting effect is mediated by central processes involved in personal space perception.

### Comparison with spatial plasticity induced by other sensori-motor manipulations

Both short and long-lasting distortions of the perceived SA have been demonstrated in healthy adults following prismatic adaptation when used as an artifice to provoke reorganisation of spatial processing [6], [7], [8]. The LP task involved no conflict and was brief when compared to the 75-minutes of viewing through prisms used by Hatada and collaborators [6], nevertheless, the biases observed in the SA direction one day after the LP task (−2.50° for the leftward pointing group and +3.96° for the rightward one) are comparable to those to be observed one day after prismatic adaptation (2.8°) [6]. Thus, major distortions in the spatial properties of a sensory input are not necessary for the development of long-lasting changes of the perceived body axes.

An interesting point of comparison of our findings with those of prism adaptation concerns the directional deviation of our SA estimates. Unlike the spatial directional bias induced by prismatic adaptation [1], [8], [17], the deviation reported here is in the same (instead of opposite) direction to our LP modulating task. However, we believe that both the prismatic adaptation and the LP task effects are not contradictory since leftward and/or rightward prismatic adaptation can be considered not only as a distortion of the sensori-motor coordination by shifting the surrounding visual field, but also as a procedure causing a lateralisation of the motor behaviour [18], [19], [20]. Such a lateralized motor component of prismatic adaptation may contribute to inducing plasticity of spatial perception. In other words, we argue that the leftward prismatic adaptation parallels our rightward LP task and rightward prismatic adaptation parallels our leftward LP task.

In favour of this comparison, our results suggest a possible direction-specific effect of the LP task, as shown for the prismatic adaptation [8], [21], [22]. During the late-test, the amplitude of the lateral bias shown by participants of the right LP group appears to increase compared to post-test. In the same vein, observation of individual data seems to indicate more systematic effects among members of the rightward LP group as compared to those of the leftward LP group. The well-known, direction-specific effect of the prismatic adaptation [1] seems to be coherent with this proposed comparison since the plasticity of spatial perception was only observed following adaptation to leftward visual shifts and not for rightward ones among healthy adults [22]. Although our results cannot decisively resolve this phenomenon and its underlying processes, they suggest that pre-existing rightward preference for action could induce more systematic and long-lasting plasticity of spatial perception than pre-existing leftward preference. This potential direction-specific effect of the LP task is worthy of further study because of striking similarity to the well-established observation that left-sided neglect is more robust and durable as compared to the right-sided disorder [9], [23], [24], [25]. The paradigm also promises insights for the understanding of the core mechanisms of spatial perception and cerebral lateralisation.

A further significant finding for lateralisation is the fact that our experiment which seems to produce biases of spatial perception that become greater and more systematic with time involved right finger movements in the right hemispace. This phenomenon is consistent with Robertson and North's results [26] who obtained a similar but inverse phenomenon in neglect patients showing that only left finger movement in left hemispace reduces neglect. This parallel should be clarified further by comparing both the current leftward and rightward LP task performed with the right index finger with the same conditions performed with the left index finger.

### Mechanism of long lasting spatial modification

Following Hatada and colleagues [6], we could explained the long-lasting influence of the LP task on space perception, as the result of a context-specific learning, or as a sleep effect and/or due to neural plasticity. Firstly, it has been suggested that the after-effect observed following prismatic adaptation could be dependant upon the similarity of context between exposure and post-exposure to prisms [18], [19], [20]. For instance, the similarity of apparatus, task context and speed of target pointing are elements which may cause the retrieval of the exposure context and, in turn ‘reactive’ the sensori-motor behaviour that has developed during exposure. This explanation is amenable to our study. However, we believe that our procedure prevented any context-dependant effect since the LP task shares little task context with the SA pointing. Unlike the LP task, which consisted in pointing towards visual targets displayed in the fronto-parallel plane, involved 3-Dimensional spatial movement and had speed instructions, the SA pointing consisted in pointing toward subjective (imagined) midsagittal point in the horizontal plane, without visual feedback, involved a 2-Dimensional spatial movement and had no time limit.

The effect of sleep is also a potential factor contributing to the significant long-lasting influence of the LP task since late-test was performed after the first post-exposure sleep period. Convergent evidence suggests that sleep is favourable for plastic cerebral changes that underlie learning and memory [see 27], [28 for review]. Sleep could facilitate the consolidation of the memory traces which, before sleep periods would remain fragile. For instance, behavioural data have shown correlation between improved performance in learning tasks performed following sleep periods and specific sleep variables (e.g., duration of the REM (rapid eye movement) and non-REM sleep). Specifically relevant to our study, Laureys and colleagues [29] have suggested that sleep could play a role in the refinement of the visuo-motor neural network related to new environmental conditions. This conventional view could explain the increased amplitude of the SA deviation assessed at the late-test as compared to immediate post-test observed in the current study for the rightward LP group and sometimes reported in others studies using prismatic adaptation [8], [30]. However, the facilitation effect of sleep in memory consolidation and neural plasticity remains elusive [27], [28] and more studies are needed to probe how plastic change occurring during a night of sleep contributes to plasticity of spatial perception observed one day after normally coordinated sensori-motor experience.

### General implications for spatial perception

Whatever the underlying neuronal processes of such long-lasting effects, we believe that the present study has important implications about how historical experiences of spatial behaviours determine spatial perception. The current results significantly generalise on recent data revealing that LP task can elicit subsequent short-term lateral deviations in line bisection towards the previously pointing hemispace, as assessed by both the visual and visuo-motor bisection tasks [12]. In overview, it was already established that the perceived geometry of ‘extracorporeal’ visual space could be affected by directional coordinated visuo-motor experiences. The present study further reveals that coordinated lateralized visuo-motor experience can also affect proprioceptive personal space as assessed through the SA pointing task. Together, these studies suggest that sensori-motor experience could partially determine multimodal (*i.e.*, visual and proprioceptive) and/or multi-represented (*i.e.*, extracorporeal and corporeal) space. More particularly, we argue that spatial perception could be influenced by our past experience about the spatial direction and/or localisation of the sensory-motor interaction with environment.

To our knowledge, these findings showing the influence of a spatial component of sensori-motor experience on space perception have been neither demonstrated nor anticipated specifically by theory. Nevertheless, this proposal sits well with the body of experimental evidence and theorisation on the role of action in perception. According to Gibson's ‘ecological approach’ [31], the environment is perceived through its potential behavioural interaction relative to the organism's physiology and ways of life. The involvement of motor action in perception is also emphasised by Rizzolatti and its colleagues [e.g.], [see 32], [33,34] who described the fascinating presence of ‘motor’ and ‘perceptive’ response in the same neurons. They suggest that the discharge in such sensori-motor neurons is likely to code a potential motor action which is accessed automatically (in the case of sensorial stimulation) or voluntarily (in the case of action execution) [see also 35], [36].

Given that potentials for action are not uniform through space, it has been proposed that space is subdivided into sectors (e.g., near peri-personal and far extra-personal space) whose the boundaries were determined by the nature of potential motor action [37], [38]. Objects can be grasped and manipulated in the peri-personal space whereas they can be thrown, reached throw locomotion or only observed when they are in the extra-personal space. It suggests that spatial perception, closely linked to the organization of action, is differently coded and or processed in these subspaces. In favour of this proposal, Coello and colleagues [39] recently provided the first evidence for brain motor areas involved in the specification of the perceived boundary of peri-personal space. Following the theme that the potential for motor action is involved in space perception, Proffitt and colleagues [see 40 for review] have studied perceived orientation (geographical slant, [e.g., 41]) and egocentric distance or extent (to a target from an observer, [e.g.], [42,43]) addressing how perception is influenced by the opportunities of acting in the space and their associated cost (e.g., the energy cost of locomotion in this space). They show for instance that wearing a heavy backpack increases perceived egocentric distance compared to a control no-backpack condition, probably due to the increase of the metabolic cost associated with walking this distance [42]. Overall, the body of these whole experiments have shown that space perception is a malleable construction partially determined by our past experience about the nature (e.g, grasp, locomotion) and the cost of our active behaviour, which in turn influences to some degrees our ever-changing potential to act on the environment and the perceived costs associated with possible actions.

The current study introduces an important new factor to the role of action in perception by showing that space perception is also influenced by the spatial properties of our past sensori-motor experience. The influence of on-going lateralized action on spatial perception has already been demonstrated, showing for instance, the influence of the directional arm movement during the task on neglect signs [26], or the influence of the starting position of the hand on straight-ahead estimation [2], [44]. To this we add the notion that perception of directions in space also tends to be warped towards the directions of actions previously performed in space. Accordingly, the geometry of spatial perception depends on the geometry of action space. As a consequence, perceived middle of personal and/or extrapersonal space could be displaced toward the action space developed through our previous sensori-motor experience and/or habits. In overview, spatial perception appears as a dynamic and malleable construction partially dependent upon the nature, the cost and the direction and/or localization of previous sensori-motor spatial interaction with environment. This new perspective of space perception could explain the phenomena that some spatial tasks are influenced by ecological experiences of behaviour which emphasises laterality, including directional preponderances in reading and writing in different languages [45], [46], [47], [48].

These results contribute to understanding the core mechanisms of spatial perception and offer insights into the plasticity of spatial perception. Further studies are indicated to investigate the interaction between normal sensori-motor processing and spatial perception in detail. More particularly, it would be interesting to compare, in the same experiment, the long-lasting dynamic of the deviation in egocentric direction following normally coordinated sensori-motor experience to the effects induced by prism adaptation. Additional studies would also be needed to test the transfer of the observed long-lasting effects to other spatial tasks and to examine the interaction between the directional component of both the previous and on-going sensori-motor activity. Indeed, our data could be interpreted to indicate that the well-known influence of directional exploration of space on SA perception (as shown by the effect of the starting point of the hand for SA pointing) [2], [44] could be further modulated by the (right and left) LP task. Finally, if our experimental procedure induces long-lasting influence on spatial processing among healthy adults, we can hypothesize that it could be effective in reducing Unilateral Spatial Neglect and with a long-term duration of improvement. Further research is thus warranted to investigate the possible transfer of our results to neglect patients and specifically to determine whether a leftward biased sensory-motor, spatial interaction with the environment is able to improve the spatial biases exhibited by patients with left sided neglect.

## Materials and Methods

### Participants

Twelve healthy left-to-right females readers (mean age: 21, SD: 6.54) volunteered to participate in the study. They had normal or corrected-to-normal vision and were right-handed, as assessed with Dellatolas et al. Questionnaires [49]. This experiment was approved by the local ethic committee (Comité d'éthique du LPNC de l'université de Grenoble II et du CNRS) and was conducted with the understanding and the written consent of each participant who were naive with regard to the precise purpose of the study.

### Materials and Procedure

**Figure 3:**
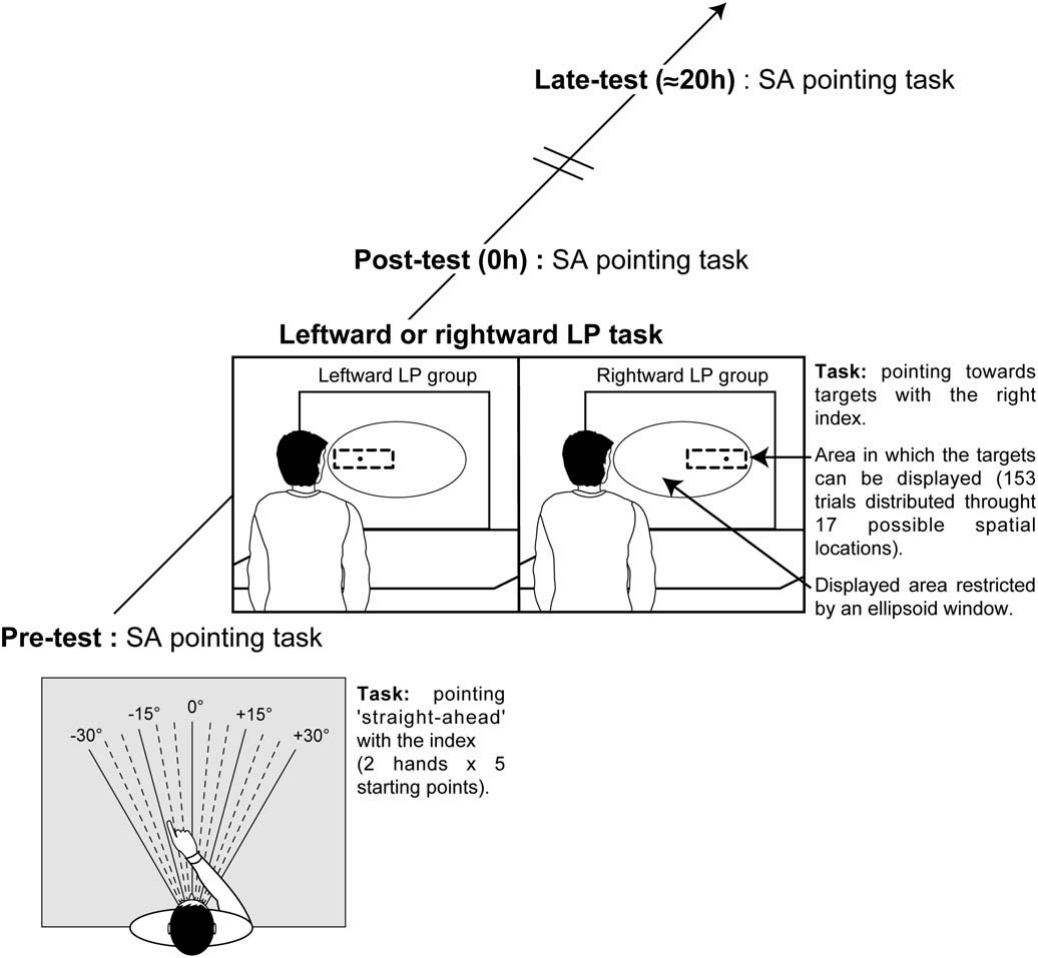
A schematic representation of the experimental design and apparatus. For the SA pointing task, the graduated values enable the experimenter to place the arm of the participants at one of the five starting positions (−30°, −15°, 0°, +15°, +30°) and to record the direction of SA to within half a degree at each trial.

Participants were tested individually and underwent a similar procedure composed of the three sessions in which they performed the proprioceptive SA task immediately before (‘pre-test’), immediately after (‘post-test’) and one day after (‘late test’, i.e., after a delay from 19 to 24 hours) the LP task (see Fig. 3 for a schematic of the procedure). Participants performed all theses tasks in front of the table, at a 600 mm distance from the computer screen used for the LP task.

For the proprioceptive SA task, participants were seated blindfolded in front of the large graduated cardboard, whose the surface was smooth to minimise tactile landmarks and were asked to point ‘straight-ahead’ with their left forefinger for half of the trials and their right one for the remaining half. Before each trial, the participant's arm was positioned at a starting point, from which they attempted to point ‘straight-ahead’, moving the arm along the table with the tip of the forefinger always in contact with the table's surface. Trunk and head positions were carefully monitored by the experimenter throughout the task so that the graduated cardboard remained centred on the sagittal midline of participants. Two trials were conducted in each of the five hand starting positions (−30°, −15°, 0°, +15°, +30°) and with each hand, yielding a total of 20 trials. There was no time limit and the finger position was recorded by the experimenter at each trial when the participant judged that his/her finger had reached and was pointing ‘straight-ahead’. The task duration was approximately 10 minutes. The apparatus enabled the direction of SA, to within half a degree. Leftward and rightward deviation from the true SA was respectively coded as negative and positive value.

During the LP task, the apparatus used for the SA task was hidden under a large cardboard sheet. One hundred and fifty three visual targets were displayed on a computer screen (376.5×300 mm, 1280×1024 pixels, 75 Hz) mounted in the fronto-parallel plane in front of the individual's head (at a distance of 600 mm) and centred on the mid sagittal line of the body. Vision of this display area was restricted by an ellipsoidal window (365 mm×270 mm) centred on the computer screen in landscape orientation, so that it blocked the black screen border as well as the surrounding equipment. Targets, which consisted of black dots of twenty pixels i.e., approximately 6 mm in diameter, were presented on a white background behind this ellipsoidal window and aligned with its horizontal axis. The targets were displayed one at a time, each for 1500 ms (with an Inter-Stimulus Interval of 300 ms) in the left or right side of the computer screen (depending on the group in which participant was assigned) and distributed across seventeen spatial locations spaced at 11.8 mm horizontal intervals. A mask (14.7×9.4 mm), built by random juxtaposition of black and white pixels, was displayed for 300 ms in order to erase the previous target. Participants were to point as quickly and as accurately as possible to each target with their right index finger and were asked to replace their hand on the table in front of them after each pointing movement. This speed instruction was constrained by the rapid presentation of the targets. This choice in the procedure allowed us to increase the difference between the procedure of the SA pointing (slow pointing with no time limit) and that of the LP task (fast repetitive pointing due to rapid target presentation) in order to reduce the probability of a task-context dependent artefact (see the second section of the discussion). The task duration was 5 minutes. A miniature patch-like device, used to prompt the participant to perform the task correctly, was placed upon the right index finger tip to lead the participant to believe that the movement parameters were recorded together with pointing accuracy. However, no measure was actually recorded during this task. In fact, the interest was in the subsequent effect of the LP on the SA performance but not in the LP performance *per se*.

### Data analysis

A three way ANOVA with LP Group (leftward pointing *vs.* rightward pointing) as a between-subject factor and Session (‘pre-test’, ‘post-test’ *vs.* ‘late-test’) and Hand (left *vs.* right) as within-subject factors was conducted on SA algebraic and absolute direction averaged for each subject across the 10 trials of each within-subject condition (Hand x Session). Trend analyses were performed when expected interactions were significant. We choose to conduct trend analyses (decomposition of the effect in its degree of freedom components) instead of pairwise comparisons because analysis of the effect of each factor with other factors held constant is not appropriate for identifying interactions [50, p. 53]. All analyses were performed using STATISTICA v5.5 and 8.0 and the alpha was defined at .05.
